# Identification and validation of reference genes for gene expression analysis in *Aphidius gifuensis* (Hymenoptera: Aphidiidae)

**DOI:** 10.1371/journal.pone.0188477

**Published:** 2017-11-30

**Authors:** Xue-Ke Gao, Shuai Zhang, Jun-Yu Luo, Chun-Yi Wang, Li-Min Lü, Li-Juan Zhang, Xiang-Zhen Zhu, Li Wang, Jin-Jie Cui

**Affiliations:** State Key Laboratory of Cotton Biology, Institute of Cotton Research, Chinese Academy of Agricultural Sciences, Anyang, Henan, China; Kumamoto University, JAPAN

## Abstract

Reference genes have been utilized in estimating gene expression levels using quantitative reverse transcriptase-quantitative polymerase chain reaction (qRT-PCR) analysis. *Aphidius gifuensis* Ashmaed is one of the most widely used biological control agents for aphids. The biological properties of this species have been studied in detail, and current investigations are focused on elucidating the regulatory mechanisms in its host However, the appropriate reference genes for target gene expression studies have not been identified. In this study, the expression profiles of 12 candidate reference genes were evaluated under different experimental conditions(development stage, sex, tissue type, diet) by using dedicated algorithms, including geNorm, Normfinder, BestKeeper, and ΔCt. In addition, RefFinder was used to rank the overall stability of the candidate genes. Finally, we recommend three optimal reference genes for the normalization of qRT-PCR data in the presence of specific variables, which include *ACTB*, *RPL13*, and *PPI* for different developmental stages; *RPS18*, *ACTB*, and *RPL13* for sexes; *RPL13*, *PRII3*, and *RPS18* in different tissue types; and *RPL13*, *RPL27*, and *ACTB* in diverse diets. The present study has identified optimal reference genes that could be used in estimating the expression levels of specific genes under these conditions following the Minimum Information for publication of Quantitative real-time PCR Experiments (MIQE) guidelines, which would facilitate in advancements in functional genomics research on *A*. *gifuensis*.

## Introduction

Real-time quantitative reverse transcriptase-polymerase chain reaction (qRT-PCR) is the most sensitive and accurate method for determining mRNA expression levels of target genes under different experimental conditions and is commonly used to confirm the expression of relevant genes in high-throughput sequencing[1–3]. To accurately estimate gene expression levels, internal reference genes are used to normalize quantitative fluorescence data on the target gene. Most internal reference genes must maintain a high degree of uniform expression at different developmental stages, environments, or experimental conditions [4]. Housekeeping genes are commonly used as internal controls because of their relatively stable expression regardless of changes in the environment, physiological conditions, and cell type, and their levels directly represent the number of cells or genomes present in the sample [5, 6].

Screening for internal reference genes has been extensively performed in various insect species and their natural enemies. *Acyrthosiphon pisum*, *Lipaphis erysimi*, *Spodoptera litura*, *Bemisia tabaci*, and *Coleomegilla maculate* are some of the commonly used internal reference genes for insects [5, 7–10]. However, numerous studies have shown that the expression levels of these housekeeping genes significantly vary under certain conditions and are thus not suitable as internal reference genes [11, 12]. Although qRT-PCR can be used as a rapid and reliable method for detecting and quantifying gene expression in different biological processes, some internal reference genes exhibit considerable differences in expression among various treatment settings [11]. Therefore, the evaluation of housekeeping genes under different experimental conditions and the selection of the appropriate gene for normalization of expression is critical.

*A*. *gifuensis* is the most abundant aphid parasitoid in the open cotton field. However, to control *A*. *gifuensis*, it is necessary to establish a standardized qRT-PCR procedure [13] that follows the Minimum Information for publication of Quantitative real-time PCR Experiments (MIQE) guidelines for *A*. *gifuensis*. The objective of the present study was to identify and validate the most suitable reference gene(s) for gene expression profiling in *A*. *gifuensis*, particularly those that are stably expressed under different treatments. The following 12 housekeeping genes were selected as candidate reference genes: dimethyladenosine transferase (*DIMT*), *ACTB*, 60S ribosomal protein L3 (*RPL13*), peptidylprolyl isomerase (*PPI*), *TUB*, *RPL18*, *18SrRNA*, *AK*, *EF1A*, *TBP*, RNA polymerase II (*PRII*), and ribosomal protein L27 (*RPL27*). Specifically, we evaluated the stability of the above candidate reference genes under different experimental conditions, including different developmental stages, sex, tissue types, and diet treatment. The expression stability of these genes was evaluated using five statistical algorithms, geNorm[14], NormFinder[15], BestKeeper[16], deltaCt method [17], and RefFinder[18]. To the best of our knowledge, this is the first report on the comprehensive evaluation of reference genes in *A*. *gifuensis*. This report thus recommends different reference genes based on each experimental condition.

## Materials and methods

### Insect materials

*A*. *gifuensis* was collected from the Institute of Cotton Research (IRC) of CAAS (Anyang, Henan, China). The adults were reared in the laboratory on cotton-melon aphids; rearing conditions were 24 ± 1°C, a 14:10 h light/dark (L:D) photoperiod, and 75 ± 5% relative humidity (RH). Different developmental stages, two sexes, different tissues in both adult males and females, and different diets were tested in various *A*. *gifuensis* samples to evaluate the stability of the candidate genes. The developmental stages included three-day-old larvae (collected at the third day after parasitized cotton aphids and dissected under a microscope), pupae, and adults (including both females and males). For sex, 30 adult females and males were collected, respectively. The tissue types included the head, thorax, and abdomen, which were dissected from various *A*. *gifuensis* adult males and females, and detected respectively. For diet, adults were maintained on pure water and honey, respectively, and then harvested at different time points, namely, 1 d, 2 d, 3 d, and 4 d. All collected samples were preserved in 1.5-mL centrifuge tubes, flash frozen in liquid nitrogen, and then stored at -80°C until RNA extraction. Each treatment was repeated three times independently.

### Total RNA extraction and cDNA synthesis

Total RNA was extracted from *A*. *gifuensis* using the SV Total RNA Isolation System (Promega, USA). RNA concentration was assessed by measuring ultraviolet absorbance at wavelengths of 260 nm (A260) and 280 nm (A280) using a Nanodrop2000C spectrophotometer (Thermo, USA). The A260/A280 ratio was maintained within the range of 1.80–2.00 to ensure mRNA integrity. The concentration of total RNA was normalized to 500 ng/μL. First-strand cDNA was synthesized using the PrimeScript^®^ RT Master Mix (Takara, Japan). The synthesized cDNA samples were stored at -20°C for later RT-qPCR analysis.

### Identification of reference genes in *A*. *gifuensis*

Candidate reference genes segments were identified from the *A*. *gifuensis* RNA-seq transcriptome dataset that was constructed by our laboratory. Primers were designed using Premier 5.0 (S1 Table) (Premier, USA) and used for cloning cDNA sequences that encoded the open reading frames of the reference genes. The conditions for PCR amplification were as follows: 35 cycles of 98°C for 10 s, 55°C for 15 s, and 72°C for 30 s. The PCR products were cloned into a T blunt simple vector (TransGen Biotech, Beijing, China) and then sequenced. Accession numbers were obtained upon submission to NCBI. A total of 12 candidate reference genes were selected as internal controls for qRT-PCR.

### Candidate reference genes and primer design

Upon confirmation of the sequences of each of the 12 candidate reference genes, primers for the subsequent RT-qPCR analyses were then designed by using the Beacon Designer 7 software (Table 1). qRT-PCR was performed using Mastercycle ep realplex (Eppendorf) following the manufacturer’s instructions. Each 20-μL reaction in every well consisted of the following: 1 μL of cDNA, 0.8 μL of each the forward and reverse primers (10 μM), 10 μL of 2× SYBR Green Premix (Promega, USA), and 7.4 μL of ddH2O. RT-qPCR reactions were conducted at the following conditions: 95°C for 2 min, followed by 40 cycles of 95°C for 15 s and 60°C for 60 s. Every template was amplified in triplicate. To verify the specificity of the gene amplifications melting curve analysis was performed. The relative standard curves of the transcripts were generated using a five-fold serial dilution of the cDNA. The corresponding qRT-PCR efficiencies (E) were calculated according to the following formula: E = (10[-1/slope] - 1) × 100.

**Table 1:** Primers used for studying reference gene expression in *A*. *gifuensis* by qRT-PCR.

Gene	Primer sequence (5’-3’)	Product length (bp)	Primer efficiency (%E)	Regression coefficient (R^2^)
**DIMT**	F:AAGCAGCAATAAGACCATC R:TCTAATTCAGCAACCATACG	133	91.56	0.9950
**ACTB**	F:GCTGTTGTGGTGAATGAG R:CAATCTATGAAGGTTATGCTCTT	121	93.04	0.9943
**RPL3**	F:TGATTGATGTTATTGGTGTTAC R:GAATGATACTCTGCTTGGAT	146	92.63	0.9941
**PPI**	F:CAAGACGTGAACCAGAAGA R:TGCTGTATGTATTATTGCTGTATC	143	93.61	0.9803
**TBP**	F:CAATAATGCCGCTTCATC R:ACTTCATCCAGGTGTTAC	184	93.04	0.9711
***RPII3***	F:CTTGTGAGGCTCTTGATTC R:GAGGCGAGGTAAAGTGTA	75	88.96	0.9952
**18SrRNA**	F:CTATGAGTCTGGTAATTGGAAT R:GCAACAACTTAAATATACGCTAT	123	95.76	0.9880
**RPS18**	F:GGTTAGCGATGATAGTTACAAT R:CAACATAGATGGCAACAGAA	167	92.63	0.9921
**AK**	F:CTTGTCTGTCTTGCTGAA R:CGATTCTGGTGTTGGTATT	112	96.2	0.9893
**EF1A**	F:AACAACCAACACCAACAC R:TAGGCATACCACGACTTC	153	90.37	0.9773
**RPL27**	F:CTGATACCAATGTCCACAA R:CCACCACAGAATCAACTT	134	92.62	0.9911
**RPL29**	F:GGAATCAAGAAGCCAACAC R:AATCTCTGGTTACGAAGGAAT	77	92.21	0.9896

### Data mining and statistical analysis

In order to select the most stable reference gene for RT-qPCR in *A*. *gifuensis*, four Microsoft Excel software Add-Ins: geNorm, NormFinder, BestKeeper, ΔCt method, and one web statistical tool, RefFinder, were used for analysis of expression profiles of 12 candidate genes using various experimental conditions. GeNorm uses the gene-stability measure M to calculate mean pairwise variations between an individual gene and the remaining candidate genes, the value of which is inversely proportional to its stability, the recommended M value is M < 0.5 for homogeneous samples, and for heterogeneous samples is M < 1. Pairwise variation (Vn/n+1) is generally used for the accurate normalization of geNorm to determine the optimal number of internal reference genes. A Vn/n+1 < 0.15 indicates that the additional reference gene will not significantly increase the standardization, The first V-value, 0.15, was after V2/3, indicates that the two reference genes are sufficient to be normalized. NormFinder uses a model-based approach to assess the overall variations in the expression of the candidate reference genes, and a more stable expression of the candidate gene shows a lower stability value. The standard deviation (SD) of the geometric mean of the Ct values of the candidate reference genes were used by BestKeeper, and the lower the index scores, the more stable the reference genes. The delta Ct method calculates the relative expression levels of the gene pairs between one and the other, and the candidate reference genes with smaller SD values are more stable. Finally, RefFinder, a web-based analysis tool, was used to compare and ranke the candidate genes, which provides an overall ranking of the stability of the candidate genes.

### Validation of the selected reference genes

To assess the effects of various treatments on the gene expression profile of the samples, the relative expression profile of *OR49b* (odorant receptor 49b) and *GIR-NMDA2B* (glutamate receptor ionotropic, *NMDA 2B*) were evaluated at three life stages (larva, pupa, and adult). The primers used to identify and amplify *OR49b* and *GIR-NMDA2B* were listed in S2 Table. The expression of *OR49b* and *GIR-NMDA2B* were normalized with the two most stable reference genes (*ACTB*, *RPL13*) and the most unstable gene (*RPL29*). The 2-ΔΔCt method was used to calculate the relative quantification of OR49b and *GIR-NMDA2B* expression [19].

## Results

### PCR amplification and expression profiling of candidate reference genes in *A*. *gifuensis*

Twelve candidate reference genes were initially studied by reverse transcription polymerase chain reaction (RT-PCR). All amplicons were sequenced and identical to their corresponding transcripts, and all gene candidates were detected on a 2% agarose gel as a single amplicon with the desired size.

The amplification efficiency (E) values of all candidate reference genes ranged from 88.96% to 96.20%, and the correlation coefficient (R2) values ranging from 0.971to 0.995 (Table 1 and S1 Fig),based on five-fold serial dilution of the pooled cDNA and the generated standard curve of each gene.

**Fig 1:**
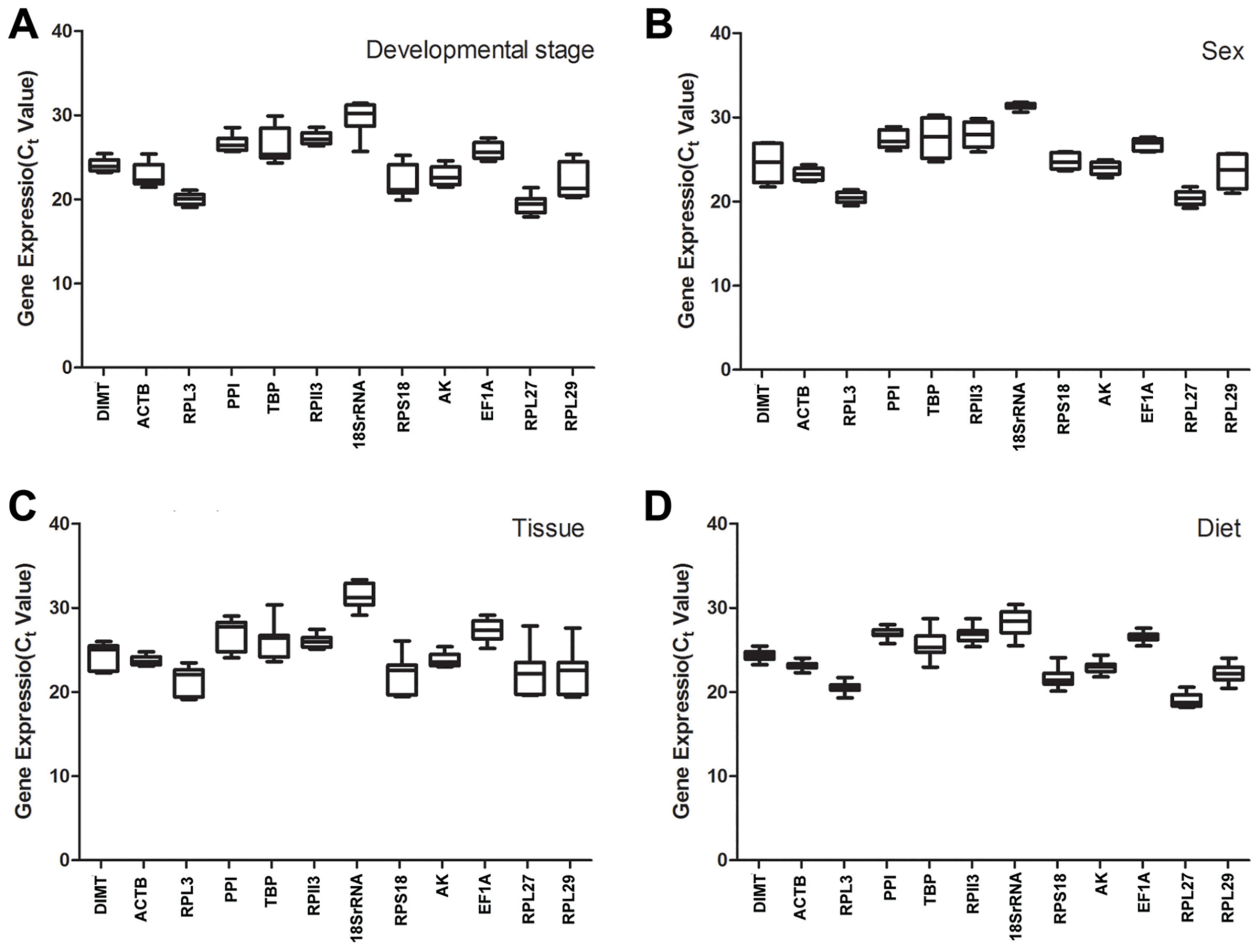
Expression profiles of the 12 candidate reference genes in all four experiments.

The levels of mRNA and the variable expression of the candidate reference genes were described using their mean values. In qRT-PCR, a variable Ct value for all the reference genes across the four treatments and the overall of all treatments indicated that their gene-expression levels were affected under different conditions (Fig 1). The expression of *TBP*, *18SrRNA*, and *RPL29* substantially varied in different development stages and sexes, whereas *DIMT*, *ACTB*, and *RPL13* and *ACTB*, *RPL13*, and *RPS18* showed the least variable expression profiles in different development stages and sexes, respectively. Additionally, almost half of the genes showed significant changes in expression levels in different tissues, whereas *ACTB*, *RPII3*, and *AK* showed a narrow range of variable Ct values. Furthermore, except for *RPS18* and *TBP* that displayed highest variations in Ct values, the range of expression values of the other candidate reference genes were very narrow. Regardless of treatments, *RPL13* performed the highest, the average Ct value was 20 in *A*. *gifuensis*.

### Expression stability and ranking of the candidate reference genes

The candidate reference genes were ranked by employing four statistical algorithms under four different conditions. Developmental stages included 3-day-old larvae, pupae, and adult females and males. For sex, adult females and males were assessed. For tissues, the head, thorax, and abdomen were dissected from *A*. *gifuensis*. For diet, H2O and honey were given to adult males and females.

#### geNorm

**Fig 2:**
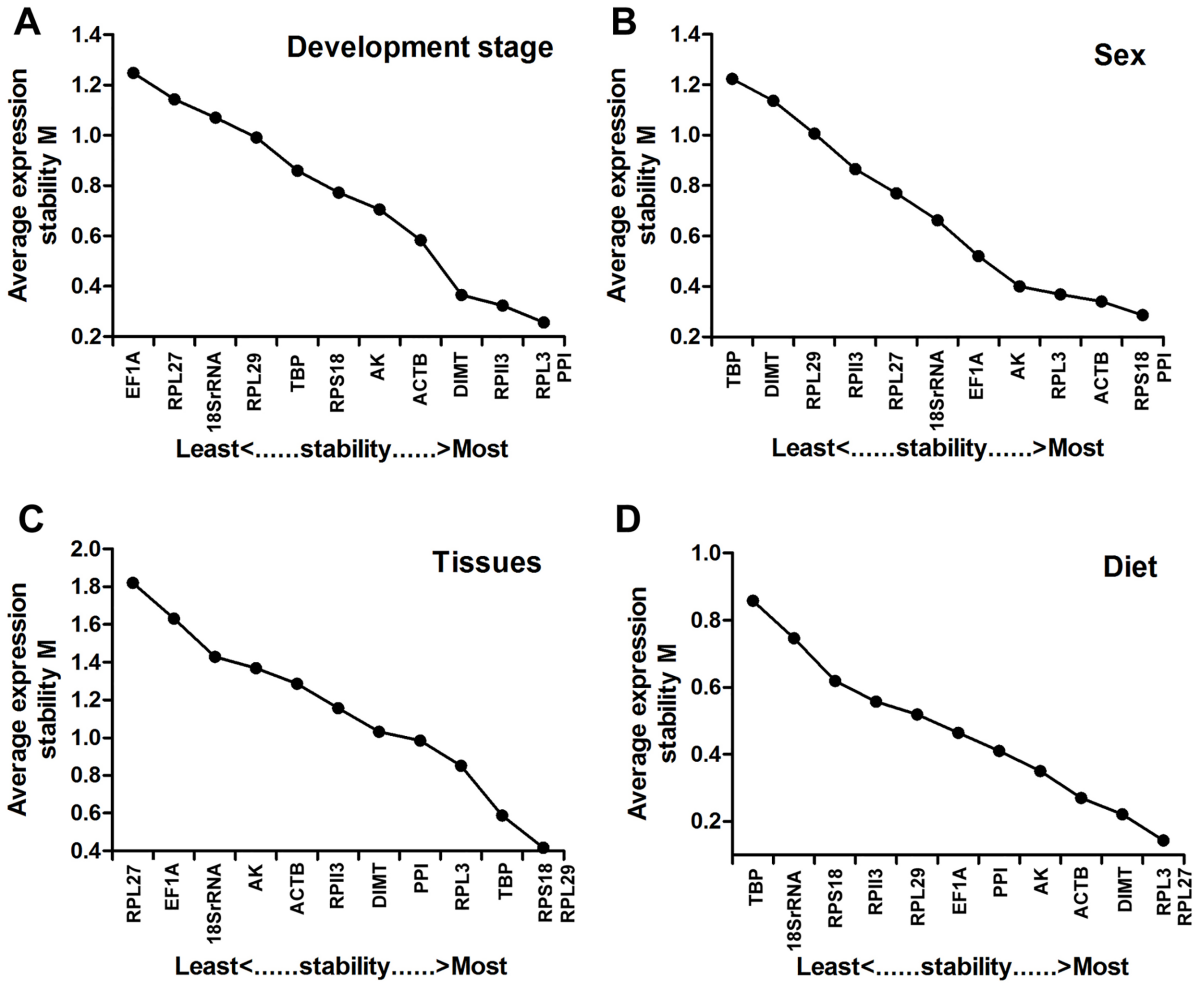
Expression stability and relative ranking of the 12 reference genes as predicted by using geNorm. The mean expression stability (M) was calculated by stepwise exclusion of the least stable gene across all the samples within a particular group set. The mean stability of different genes is plotted; the least stable genes are represented on the left and the most stable on the right side of the plot.

**Fig 3:**
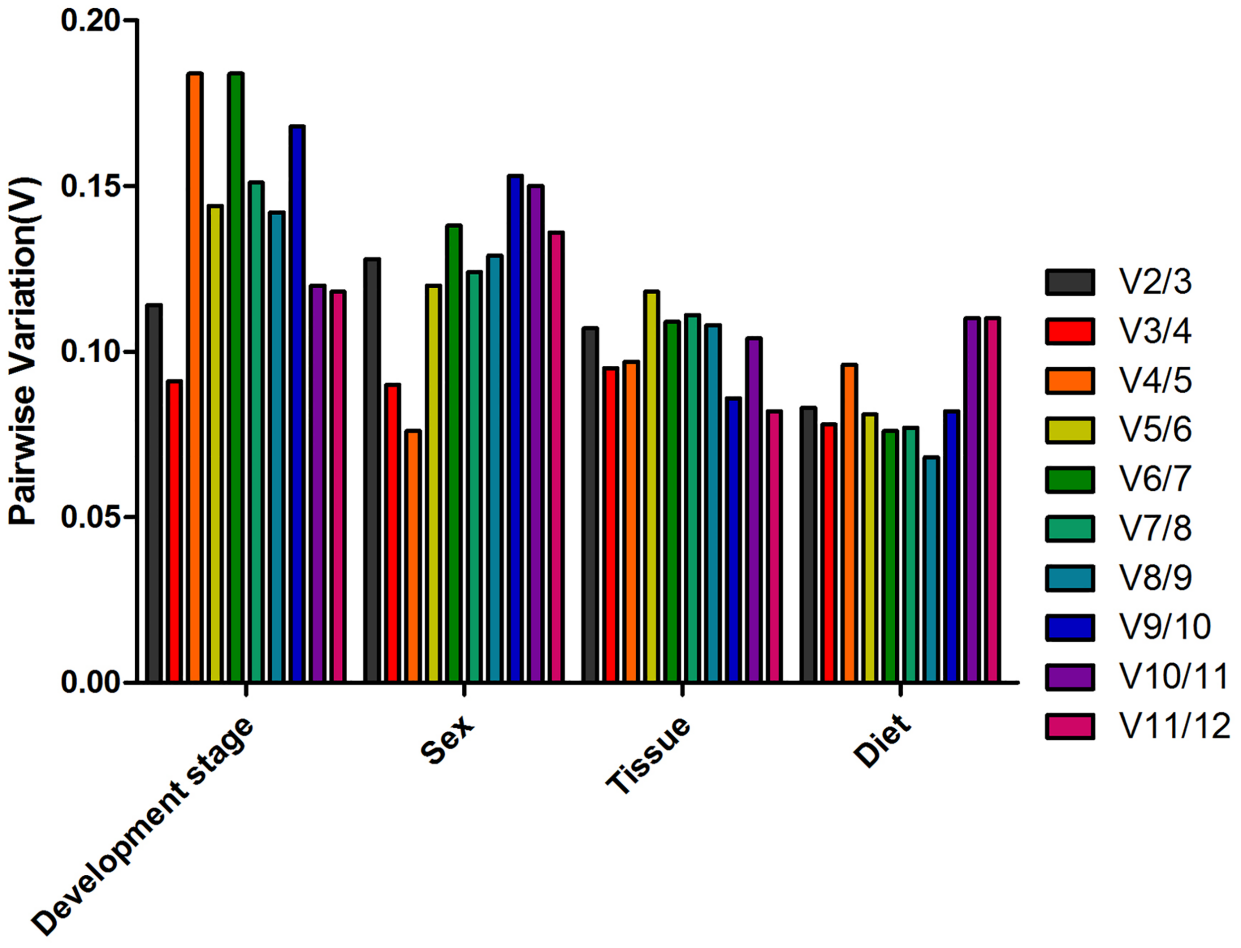
Pairwise variation (V) values in four experimental groups by using *geNorm*. Average pairwise variations (V) were calculated between the normalization factors NFn and NFn+1 by geNorm software to indicate the optimum number of reference genes required for qRT-PCR data normalization. A threshold value below 0.15 indicated that the additional reference gene has no significant improvement on normalization in qRT-PCR data.

Considering the data obtained from various treatment conditions, all the reference genes showed significant variations in their expression levels across various treatment conditions. Developmental stage analyses identified *RPL13* and *RPII3* as the most stable reference genes, whereas *RPS18* and *TBP* were the most unstable reference genes, respectively (Fig 2A). For sex, *RPS18* and *ACTB* were co-ranked as the most stable reference genes (Fig 2B). For different tissues, the geNorm demonstrated that the expression of *RPS18* and *RPL29* were the most stable, whereas *RPL27* was the most unstable (Fig 2C). The experiment using diverse diets indicated that *RPL13* and *PRL27* were the most stably expressed reference genes, followed by *DIMT* and *ACTB* (Fig 2D). The overall order based on geNorm from the most stable to the least stable reference genes were shown in Table 2. To determine the minimum number of genes required for normalization, we used geNorm to calculate the V-value. Our study showed that all the pairwise variation V2/3 values were < 0.15 across different conditions. Based on the same principle, in all treatments, two reference genes are needed for the reliable normalization with the first V-value < 0.15 appearing at V2/3 in *A*. *gifuensis* samples (Fig 3).

**Table 2:** Stability of reference gene expression under four experimental conditions.

Experimentconditions	Reference gene	geNorm		NormFider		BestKeeper		ΔCt	
		Stability	Rank	Stability	Rank	Stability	Rank	Stability	Rank
**Developmental stage**	*DIMT*	0.365	3	0.96	7	0.611	1	1.21	7
	*AK*	0.706	5	0.58	3	0.912	7	1.096	5
	*PPI*	0.323	2	0.542	2	0.741	4	1.007	2
	*RPII3*	0.257	1	0.723	6	0.621	2	1.077	4
	*TBP*	1.07	9	1.167	10	1.685	11	1.413	10
	*ACTB*	0.583	4	0.345	1	1.105	8	1.001	1
	*18SrRNA*	1.248	11	1.567	12	1.269	9	1.775	12
	*RPS18*	0.991	8	1.094	9	1.561	10	1.366	8
	*RPL13*	0.257	1	0.665	5	0.694	3	1.033	3
	*RPL27*	0.859	7	1.072	8	0.82	6	1.375	9
	*RPL29*	1.143	10	1.238	11	1.707	12	1.502	11
	*EF1A*	0.772	6	0.621	4	0.764	5	1.12	6
**Sex**	*DIMT*	1.136	10	1.474	11	2.238	11	1.57	11
	*AK*	0.369	3	0.428	4	0.622	4	0.96	4
	*PPI*	0.4	4	0.255	2	0.96	8	0.95	3
	*RPII3*	0.865	8	0.569	5	1.502	9	1.1	6
	*TBP*	1.224	11	1.587	12	2.327	12	1.66	12
	*ACTB*	0.286	1	0.349	3	0.708	6	0.94	2
	*18SrRNA*	0.662	6	1.36	9	0.289	1	1.51	9
	*RPS18*	0.286	1	0.148	1	0.822	7	0.89	1
	*RPL13*	0.34	2	0.588	6	0.542	2	1	5
	*RPL27*	0.769	7	1.371	10	0.623	5	1.54	10
	*L29*	1.007	9	1.165	8	1.923	10	1.4	8
	*EF1A*	0.52	5	0.795	7	0.62	3	1.16	7
**Tissue**	*DIMT*	1.032	5	0.721	3	1.299	5	1.502	3
	*AK*	1.369	8	1.253	8	0.689	2	1.803	8
	*PPI*	0.985	4	0.824	4	1.543	7	1.535	4
	*RPII3*	1.157	6	0.616	2	0.709	3	1.48	2
	*TBP*	0.588	2	1.17	7	1.586	8	1.712	7
	*ACTB*	1.286	7	1.105	6	0.438	1	1.683	6
	*18SrRNA*	1.428	9	1.321	9	1.151	4	1.838	10
	*RPS18*	0.416	1	1.082	5	1.809	10	1.625	5
	*RPL13*	0.851	3	0.37	1	1.416	6	1.372	1
	*RPL27*	1.821	11	2.556	12	1.978	12	2.738	12
	*L29*	0.416	1	1.37	10	1.953	11	1.804	9
	*EF1A*	1.632	10	2.529	11	1.757	9	2.735	11
**Diet**	*DIMT*	0.221	2	0.219	3	0.479	5	0.653	3
	*AK*	0.35	4	0.334	4	0.554	7	0.706	5
	*PPI*	0.41	5	0.499	7	0.518	6	0.785	7
	*RPII3*	0.519	7	0.46	6	0.725	8	0.771	6
	*TBP*	0.746	10	1.303	11	1.168	11	1.398	11
	*ACTB*	0.27	3	0.353	5	0.399	1	0.698	4
	*18SrRNA*	0.858	11	1.326	12	1.201	12	1.418	12
	*RPS18*	0.619	9	0.772	10	0.861	10	0.968	10
	*RPL13*	0.143	1	0.071	1	0.444	3	0.61	1
	*RPL27*	0.143	1	0.083	2	0.422	2	0.61	2
	*L29*	0.557	8	0.51	8	0.8	9	0.817	8
	*EF1A*	0.464	6	0.631	9	0.466	4	0.86	9

#### BestKeeper

Based on the SD of the Ct values in BestKeeper analysis, *18SrRNA* was identified as the most stably expressed gene, and *RPL29* was the least stably expressed gene in different developmental stages. For sex, *18SrRNA* was considered the most stable gene, whereas *TBP* showed the highest SD. *ATCB* was the most stable genes in different tissues and diet samples under the algorithmic principle by BestKeeper, whereas *RPL27* and *RPS18* were the least stable genes in different tissues and diet samples, respectively. The overall order of the most stable reference genes based on BestKeeper is shown in Table 2.

#### NormFinder

In our study, NormFinder analysis indicated that *ACTB* was the most stable gene in different development stages. For sex, *RPS18* showed the highest expression stability, which was similar to the result of geNorm. Among different tissues and diet, the most stable gene was *RPL13*; however, in geNorm, *RPS18* and *RPL29* were the most stable reference genes in different tissues, whereas these were identified as unstable in NormFinder. Based on the results of NormFinder analysis the overall order of the most stable to the most unstable reference gene is shown in Table 2.

#### DeltaCt method

Table 2 shows that under the inversely proportional (average SD) ΔCt method, *ACTB* was the most stably expressed reference gene in different development stages, whereas in terms of different sexes, *RPS18* was identified as most stably expressed gene. *RPL13* was the most stable gene for both different tissues and diet experiments. Based on the results of the ΔCt method, the overall order from the most to least stable expressed reference genes is shown in Table 2.

### Comprehensive ranking of reference genes

**Fig 4:**
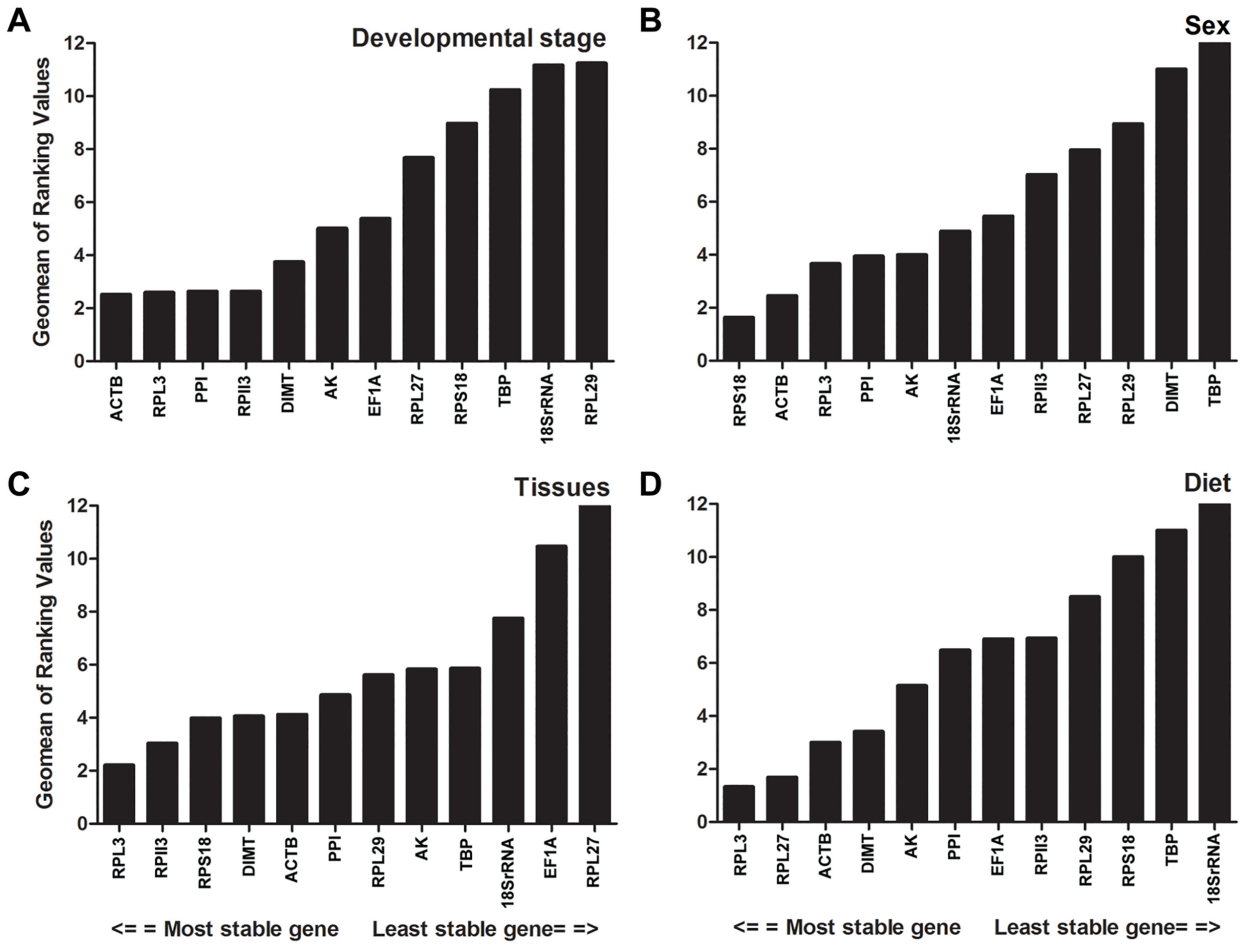
Stability of candidate reference gene expression under different treatments. A lower *Geomean* value indicates more stable expression according to *RefFinder*.

The overall ranking of 12 candidate reference genes under the four treatments was generated, based on the geometric mean (GM) of the rankings obtained from four complementary statistical methods,. The following reference genes are ranked in descending order of expression stability: different development stages: *ACTB*, *RPL13*, *PPI*, *PRII3*, *DIMT*, *AK*, *EF1A*, *RPL27*, *RPS18*, *TBP*, *18SrRNA*, and *RPL29* (Fig 4A); for sex: *RPS18*, *ACTB*, *RPL13*, *PPI*, *AK*, *18SrRNA*, *EF1A*, *PRII3*, *RPL27*, *RPL29*, *DIMT*, and *TBP* (Fig 4B); for tissue type: *RPL13*, *PRII3*, *RPS18*, *DIMT*, *ACTB*, *PPI*, *RPL29*, *AK*, *TBP*, *18SrRNA*, *EF1A*, and *RPL27* (Fig 4C); for diet: *RPL13*, *RPL27*, *ACTB*, *DIMT*, *AK*, *PPI*, *EF1A*, *PRII3*, *RPL29*, *RPS18*, *TBP*, and *18SrRNA* (Fig 4D).

### Validation of the recommended reference genes

**Fig 5:**
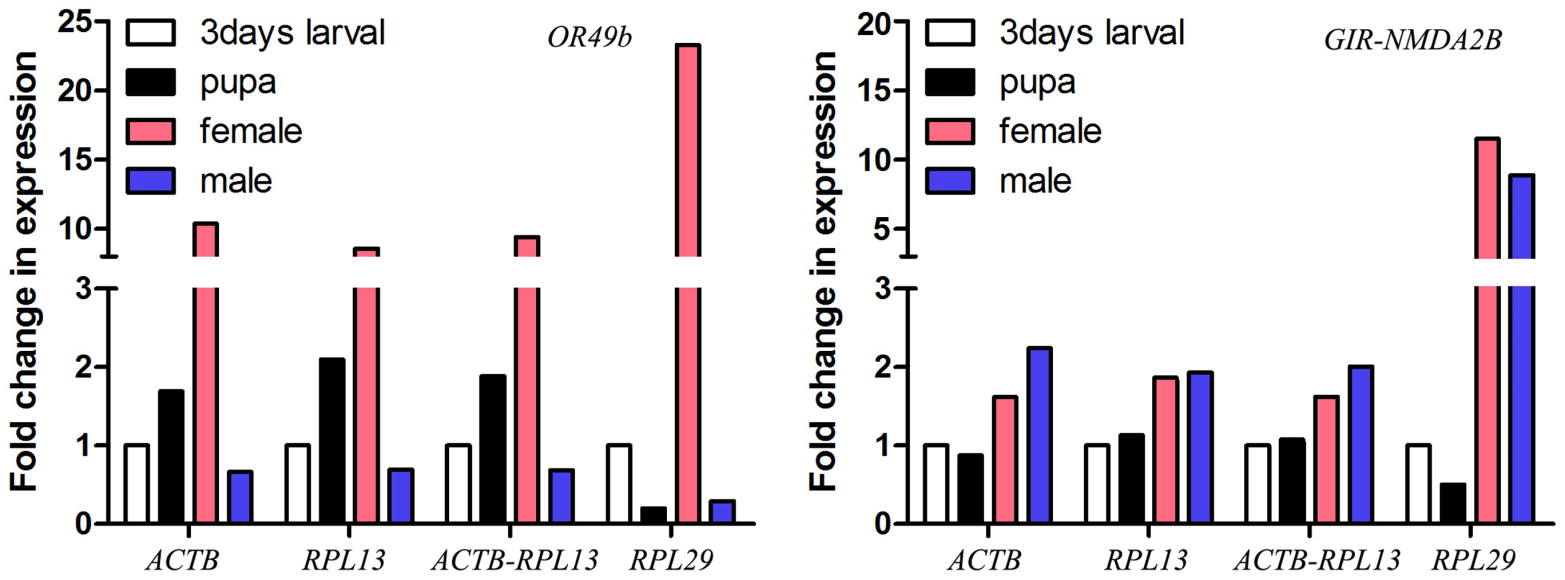
Validation of the recommended reference genes. Expression profiles of *OR49b* and *GIR-NMDA2* were investigated using different normalization factors. The expression of *OR49b* and *GIR-NMDA2* was normalized using the best reference gene (*ACTB*), the second best reference gene (*RPL13*), the top two NF (*ACTB*—*RPL13*) and the worst reference gene (*RPL29*). Bars represent the means and standard deviation of three biological replications.

Analysis of the consolidated data indicated that *ACTB* and *RPL13* were the most stably expressed gene at different developmental stages, whereas *RPL29* showed the lowest stability in expression. To examine the validity either as single or in combination of the selected reference genes, the applicability of these reference genes in normalization was tested. Odorant receptor (*OR49b*) and *GIR-NMDA2B* were investigated under different developmental stages. In the case of different developmental stages, either *ACTB* and *RPL13* or their combination as normalizer showed more consistent qRT-PCR data compared to that using *RPL29* in normalization (Fig 5). Furthermore, the expression profiles of *OR49b* and *GIR-NMDA2B* clearly exhibited differences when *RPL29* was used in the normalization.

## Discussion

QRT-PCR has become an important tool for gene expression analysis based on its high accuracy, specificity, sensitivity, and repeatability, [20–23]. However, variations in qRT-PCR data may be unavoidable during PCR analysis [6, 7], and this may be due to differences in specific conditions. Therefore, normalization using reference genes has been adapted into qRT-PCR assays to offset confounding variations among extensive experimental datasets [8, 9, 24, 25]. An ideal reference gene should be stably expressed in all test samples [26]. Unfortunately, qRT-PCR analysis is largely influenced by the choice of reference genes, and in response to different experimental conditions, the expression of commonly used reference genes may significantly change[14, 27, 28]. Thus, candidate reference genes should be validated under different conditions before utilized in actual research studies. A growing number of reference genes has been assessed for application to qRT-PCR analyses [5, 8, 10, 29–32]. However, no related studies involving references genes in *A*. *gifuensis* have been conducted to date. The present study performed a comprehensive evaluation of 12 commonly used reference genes using geNorm, NormFinder, BestKeeper, ΔCt method, and RefFinder at different developmental stages, sexes, tissue types, and diets.

Unlike previous studies [32, 33], the present study detected significant differences in candidate gene expression levels under specific conditions and based on four independent statistical analyses, which is similar to the results of previous investigations [27, 34, 35]. For example, in terms of developmental stage, *RPL13* and *ACTB* were co-ranked by geNorm as the most stably expressed reference genes, whereas BestKeeper identified *DIMT*, and NormFinder and ΔCt method indicated *ACTB*. In terms of different tissues, the four analytical approaches generated similar results, wherein *18SrRNA* and *RPL29* were the most stably expressed genes by geNorm, BestKeeper identified *ACTB*, and NormFinder and ΔCt method *RPL13*. In addition, *RPS18* and *RPL13* were identified as the optimal reference genes by geNorm, and NormFinder and ΔCt identified the same gene for sexes and diets, respectively, whereas *DIMT* and *ATCB* were the more stably expressed genes based on BestKeeper. BestKeeper has been shown to exhibit the highest number of discrepancies [36]. Based on our findings, we conclude that RefFinder generated the most reliable results because it combined the four algorithms. Therefore, the optimal reference genes for the four common variables in *A*. *gifuensis* were as follows: *ACTB*, *RPL13*, and *PPI* for developmental stage, *RPS18*, *ACTB*, and *RPL13* for sex, *RPL13*, *PRII3*, and *18SrRNA* for tissue type, and *RPL13*, *RPL27*, and *ACTB* for diet.

The most commonly used internal reference genes include actin, tubulin, and 18S ribosomal RNA (rRNA), which are integral components of cells and are thus essential for the maintenance of physiological activities[4, 23, 37–39]. The transcript levels of these reference genes are generally less susceptible to the external environment than other genes [40]. However, certain limitations such as batch to batch variations in output and variable reverse transcription and PCR efficiencies may influence threshold (Ct) values, which are the main data collected during normalization [16, 41, 42]. Collectively, the results of our study have validated the findings of earlier reports that no single reference gene is stably expressed in all treatments [8, 27, 30, 43]. The novel reference gene, *DIMT*, which encodes a dimethyladenosine transferase that, specifically dimethylates two adjacent adenosines and is situated within a conserved hairpin loop near the 3′-end of the 18S rRNA of the 40S ribosomal subunit[44], is differentially expressed among different developmental stages and sexes. The present study has also determined that *RPII3* should be excluded as a stably expressed reference gene, similar to the findings of a previous study involving Raphanus sativus L.[45]. *ACTB* is constitutively expressed and is a widely used reference gene for insects [27, 46–49]; however, the use of actin genes has recently been challenged [11]. The results of the present study also show that *ACTB* is stably expressed during different developmental stages and sexes. Ribosomal proteins are involved in translation and protein synthesis. In the present study, geNorm analysis identified *RPL13* as the most stably expressed candidate reference gene in different tissue types and diets, whereas *RPS18* was the most stably expressed gene for different sexes, and *RPL29* in different tissue types. Similar to previous studies, the findings of our study indicate that ribosomal proteins are the best reference genes for investigating expression profiles in insects [37, 50]. However, *RPL27* exhibited relatively unstable expression in our study. Furthermore, *TUB*, *EF1A*, *TBP*, and *AK*, which have been previously reported to be the most stably expressed genes [27, 37, 51], showed relatively unstable in expression using each variable. These results further suggest that the most suitable reference genes do not necessarily meet the requirement that these are expressed at constant levels under different conditions in various species [5, 6, 52]. Therefore, it is a highly recommended that a customized reference gene be selected for specific experimental conditions.

ORs are key components of insect olfaction. Odorant-binding proteins (OBPs) are transported through the infectious lymphids, filling the cavity around the dendrites and activating ORs, which in turn transform external chemical signals into signaling currents. Ionotropic glutamate receptor, which are key players in synaptic plasticity, mediate the transmission of excitatory synapses[53]. The validation of reference genes was confirmed by assessing the expression profile of *OR49b* and *GIR-NMDA2B*. The results showed that transcript abundance is strongly influenced by *A*. *gifuensis* development. The expression after normalization by *RPL29* differed from that of *ACTB* or *RPL13*. Thus, the normalized results based on *RPL29* cannot reliably reflect the expression level of target genes in *A*. *gifuensis*.

In conclusion, functional genomics and gene expression remain essential research topics relating to *A*. *gifuensis*. In this study, five algorithms (geNorm, NormFinder, BestKeeper, ΔCt method, and RefFinder) were used to evaluate the expression profiles of 12 candidate reference genes of *A*. *gifuensis* across four variable (developmental stage, sex, tissue type, and diet). We have identified the appropriate genes that can be used in the normalization of the qRT-PCR data under each variable. Furthermore, we suggest two reference genes that can be used not only to normalize expression data but also for more conservative estimates of target gene expression levels in *A*. *gifuensis*. Our findings may serve as a foundation for future genetics and functional genomics research on this particular insect species.

## Supporting information

S1 Fig Analysis showing amplification efficiency of the qRT-PCR primers. (TIF)

S1 Table Primers used in amplifying 11 reference genes in *A*. *gifuensis*. (DOCX)

S2 Table Primers used in amplifying and quantifying OR49b in *A*. *gifuensis*. (DOCX)
