# Supplementary figures and images for: Identification and validation of reference genes for gene expression analysis in Aphidius gifuensis (Hymenoptera: Aphidiidae)
Source: PLoS One. 2017 Nov 30;12(11):e0188477. doi: 10.1371/journal.pone.0188477 (PMC5708624; doi:10.1371/journal.pone.0188477)

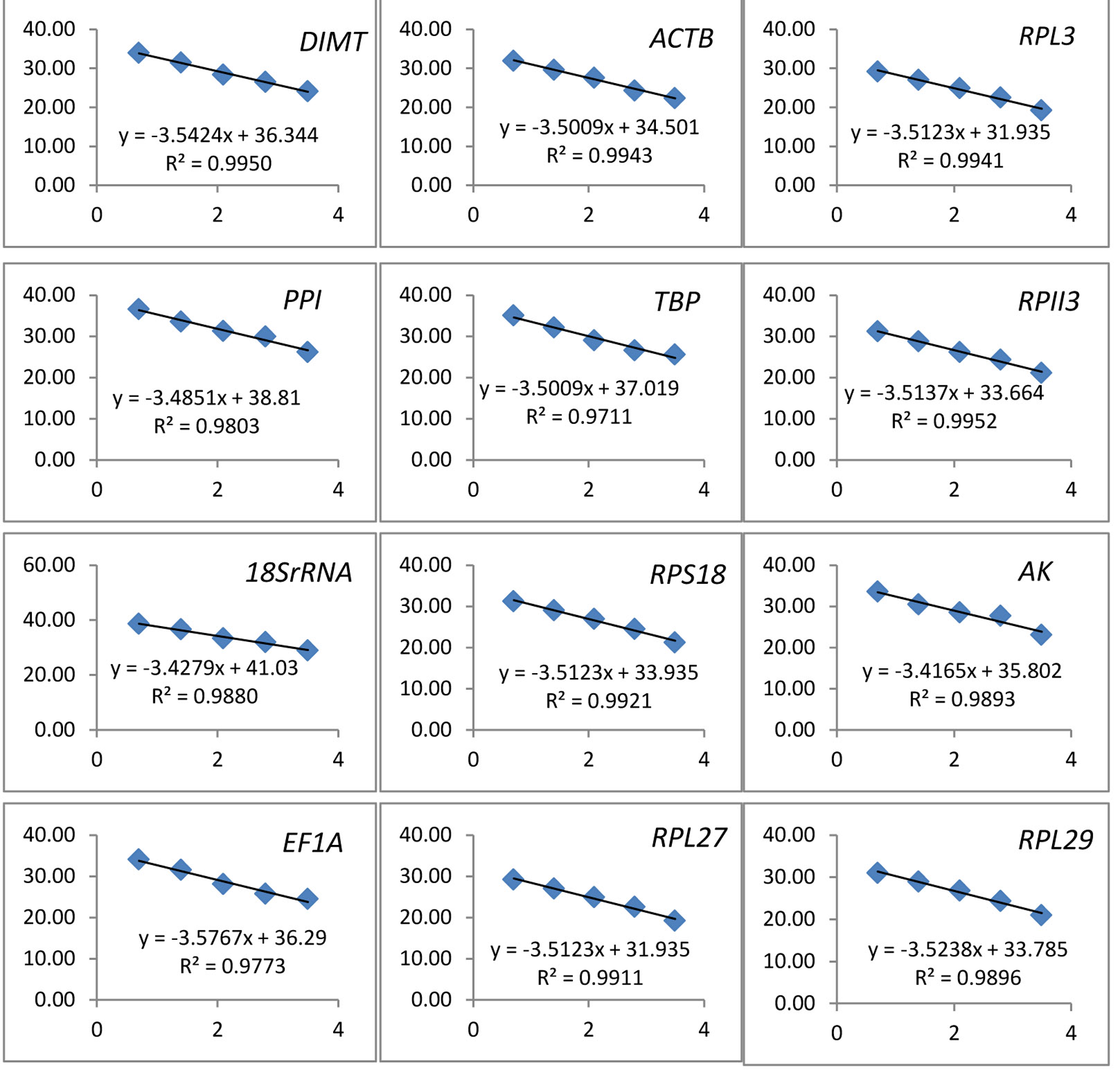

Supplement: S1 Fig — (TIF) [file pone.0188477.s001.tif]
