# Supplementary material for: Identification and validation of reference genes for gene expression analysis in Aphidius gifuensis (Hymenoptera: Aphidiidae)
Source: PLoS One. 2017 Nov 30;12(11):e0188477. doi: 10.1371/journal.pone.0188477 (PMC5708624; doi:10.1371/journal.pone.0188477)
Supplement: S1 Table — (DOCX) [file pone.0188477.s002.docx]

**S1 Table**. **Primers used in amplifying 11 reference genes in *A. gifuensis*.**

| **S No** | **Gene** | **Accession No. (*A. gifuensis*)** | **Primer sequence (5’-3’)** | **Tm (ºC)** | **Product length (bp)** |
| --- | --- | --- | --- | --- | --- |
| 1 | *Dimethyladenosine transferase (DIMT)* | KY809864 | F:CATAATAAAAATTCATAATGCCGA  R:ACATTACTTTCAACTTTTGGAGGT | 55 | 616 |
| 2 | *ß-actin*  *(ACTB)* | KY809865 | F:GTTGGCATACAAGTCCTTACGA  R:TGGAGATGACGCACCCC | 55 | 823 |
| 3 | *Ribosomal protein L3 (RPL3)* | KY809866 | F:TCATCGTGGTAAAGTAAAGGC  R:CTTCCGTCTTTGGTGTGG | 55 | 820 |
| 4 | *Peptidylprolyl isomerase (PPI)* | KY809867 | F:TGGACAAGGAACTAAAGAACC  R:AAGTGCTGTATGTATTATTGCTGT | 55 | 877 |
| 5 | *TATA box binding protein (TBP)* | KY809868 | F:CAGTCCGAGTTATCATCGCTAT  R:TACAGTTGAAGAGCAAGAAGCAC | 55 | 702 |
| 6 | *RNA polymerase II (RPII3)* | KY809869 | F:TGTTTCATCCAACGCCTT  R:TGAGAACTACCAACACTGCC | 55 | 562 |
| 7 | *18S ribosomal RNA*  *(18SrRNA)* | KY809870 | F:ATTACCCAATCCCGACACG  R:GCGACCATACTCCCCCC | 55 | 686 |
| 8 | *Ribosomal protein s18(RPS18)* | KY809871 | F:CGTCCAGTTGTCTTTGTGTG  R:GGCTTCGCACCAAACAT | 55 | 489 |
| 9 | *Arginine kinase (AK)* | KY809872 | F:AGATGTCATAAACACCACCTTCA  R:GGATAAATTGGAAGCTGGCTA | 55 | 953 |
| 10 | *Elongation factor 1-alpha (EF1A)* | KY809873 | F:TGGCGAATGTGAGGGACA  R:TGGAAGCAGTTGCGGTTG | 55 | 1698 |
| 11 | *Ribosomal protein L27 (RPL27)* | KY809874 | F:TCCACCAACAGCCTTGATTT  R:GTGGTCGTGGTAATGCTGG | 55 | 338 |
| 12 | *Ribosomal protein L29 (RPL29)* | KY809875 | F:TCGTAATGGCAAAGTCAAAGA  R:GCTTCACGACGGGCTTTT | 55 | 211 |
