# Supplementary material for: Identification and validation of reference genes for gene expression analysis in Aphidius gifuensis (Hymenoptera: Aphidiidae)
Source: PLoS One. 2017 Nov 30;12(11):e0188477. doi: 10.1371/journal.pone.0188477 (PMC5708624; doi:10.1371/journal.pone.0188477)
Supplement: S2 Table — (DOCX) [file pone.0188477.s003.docx]

**S2 Table. Primers used in amplifying and quantifying OR49b in A. gifuensis.**

| Gene | Accession No.  (A. gifuensis) | Primer sequences (5’-3’) |
| --- | --- | --- |
| *OR49b-* amplifying | KY809877 | F: TTGGAACCTTTTATCAAGTGTG |
|  |  | R: CAAACCTGTCTACTTTCGCTTA |
| *OR49b-* quantified |  | F: AATGTTGATGTTATTGACAAGAAC |
|  |  | R: TGGCATTGTAGACTGAGAG |
| GIR*-NMDA2B-* amplifying | MF737521 | F: TATGAAAATGTTTAAAGCAATTCT |
|  |  | R: ACTAACATCACAGAACAGTCTCAAT |
| GIR*-NMDA2B-* quantified |  | F: TTATGAACTACGAGCAATATGG |
|  |  | R: AATGACTGGAATACCGAGATA |
